# High Risk Features of an Anomalous Origin of the Right Coronary Artery

**DOI:** 10.1155/2021/1649723

**Published:** 2021-10-15

**Authors:** Dre Eleonore Campiche, Jean-Paul Vallée, David Carballo

**Affiliations:** ^1^Cardiology Department, University Hospitals of Geneva, 1205 Geneva, Switzerland; ^2^Radiology Department, University Hospitals of Geneva, 1205 Geneva, Switzerland

## Abstract

Anomalous aortic origin of the coronary arteries (AAOCA) is a rare congenital abnormality. It is usually asymptomatic and often found incidentally during coronary angiography. However, it can also be discovered during the autopsy of young healthy adults who have suffered from sudden cardiac death (SCD). AAOCA represents the second most common cause of SCD in young athletes. Herein, we report a case of a 39-year-old patient with left-sided right coronary anomaly with multiple high-risk features who presented with life-threatening symptoms for SCD but normal electrocardiography, echocardiography, and cardiac markers. The coronary computed tomography revealed an anomalous coronary artery from the left sinus of Valsalva with a hypoplasic origin and a high-risk path between the aorta and the pulmonary artery with a short intramural path. He was surgically managed with a coronary artery bypass with an uneventful follow-up.

## 1. Background

Anomalous aortic origin of the coronary arteries (AAOCA) is a rare congenital abnormality. It can be asymptomatic but usually found during diagnostic angiography for workup of symptoms or during acute myocardial infarctions making the management options very complicated. However, it can also be discovered during autopsy of young healthy adults who suffered from sudden cardiac death (SCD) [1]. AAOCA represent the second most common cause of SCD in young athletes [2].

Herein, we report a case of a patient with left-sided right coronary anomaly with multiple high-risk features who presented with life-threatening symptoms for SCD.

## 2. Case Presentation

A 39-year-old nonsmoking male native from Cameroun without any significant personal medical history or family history of cardiovascular disease presented to the emergency department with sudden loss of consciousness. At the end of a two-hour run ending on an uphill path, the patient felt unwell with blurred vision. This was followed by chest pain, palpitations, shortness of breath, and eventually a brief loss of consciousness.

The patient was brought to the emergency department by ambulance. On admission, the electrocardiogram showed no signs of ischemia, and the troponin T levels were slightly elevated but remained stable (15 ng/l at admission, 29 ng/l after one hour, and 15 ng/l after two hours). An acute coronary syndrome was ruled out. An echocardiography performed by the specialist revealed a preserved left ventricular systolic function with an estimated ejection fraction of 60-65% and normal left ventricular parietal kinesis. The patient was admitted for further investigations.

**Figure 1:**
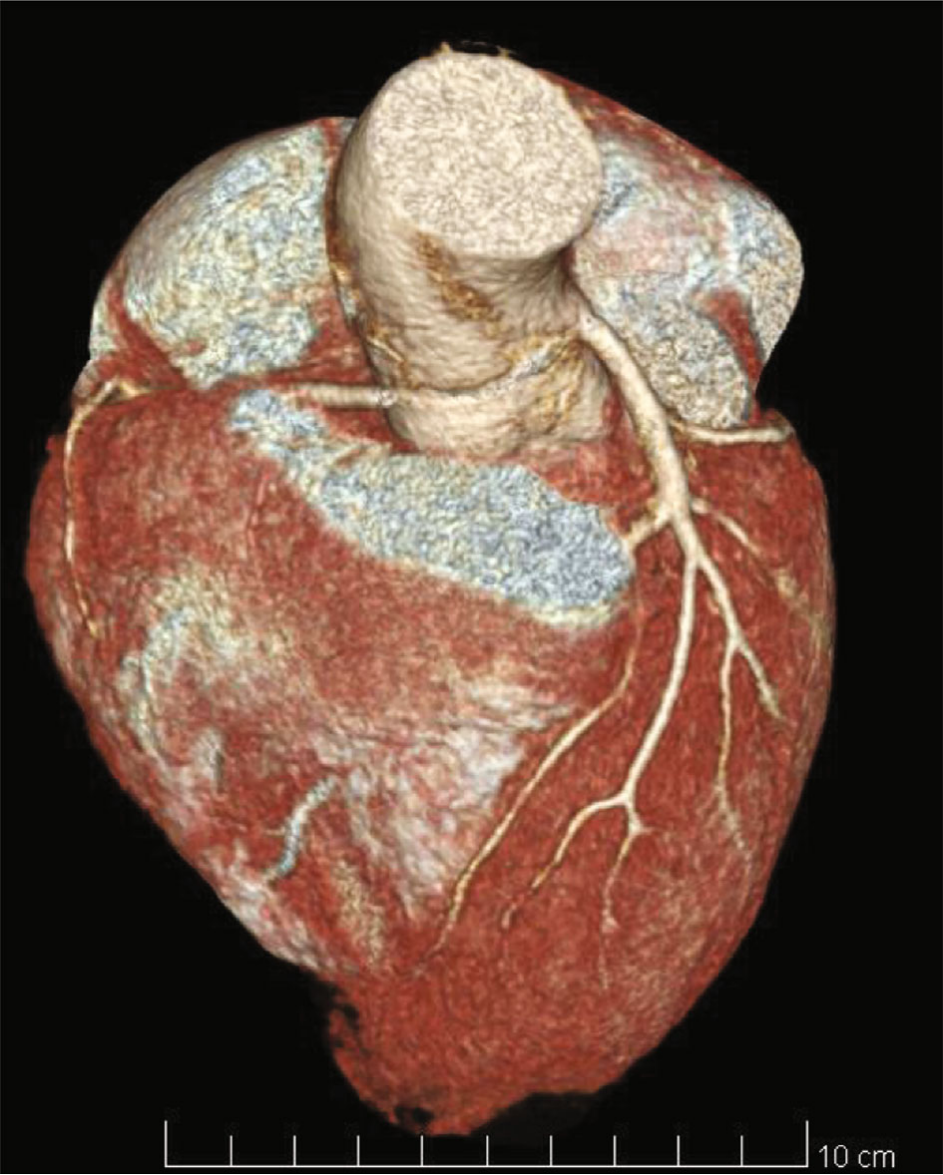
Coronary computed tomography showing the intramural path of the right coronary artery with origin close to the left coronary artery.

**Figure 2:**
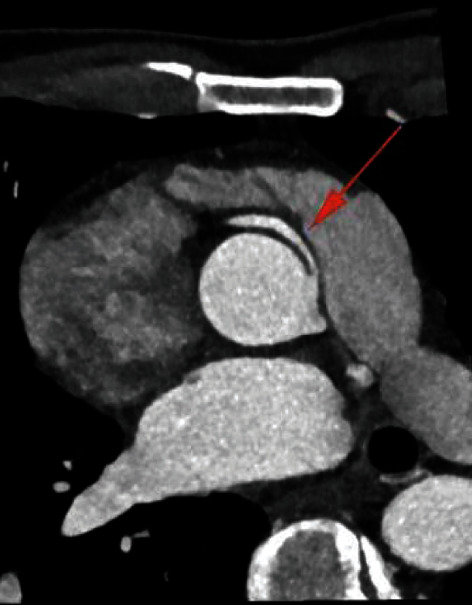
Coronary computed tomography showing the hypoplasic birth of right coronary artery (red arrow) originating close to the left sinus of Valsalva with further high-risk path between the aorta and the pulmonary artery.

**Figure 3:**
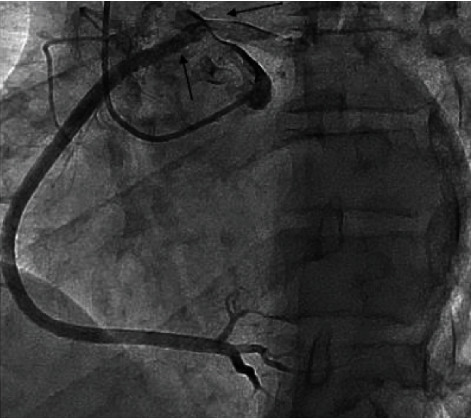
Coronary angiography revealing no atherosclerosis and the anomalous right coronary artery (left arrow) close to the origin of the left coronary artery (right arrow); with inter-aortico-pulmonary high-risk path.

A coronary computed tomography (CT) (Figures 1 and 2) showed a congenital anomaly of the right coronary artery (RCA) originating from the contralateral sinus of Valsalva, 18 millimetres above the left coronary leaflet. The right and left coronary arteries were located close to each other, but separate from the ostia; this was corroborated on the coronary angiogram (CA) which furthermore did not show any intraluminal coronary atherosclerosis (Figure 3). The origin of the RCA was hypoplasic and followed a high-risk path between the aorta and the pulmonary artery with a short intramural path. Additionally, a positron electron tomography and contrast-enhanced computed tomography (PET-CT) showed discrete ischemia during physiological stress in the basal, inferobasal, and middle lateroapical segments, representing 10% of the left ventricle in the territory of the RCA, as well as in a part of the apex segment.

After multidisciplinary assessment, the patient underwent coronary artery bypass grafting. Postoperative course was uneventful, and the patient was discharged on postoperative day 6.

## 3. Discussion

Manifestations of a left-sided origin of the RCA are illustrated by our case report. This rare anomaly can be life-threatening, as such, we aim to discuss the prevalence of AAOCA, its variants, and its high-risk features.

As previously mentioned, anomalous coronary disease is the second most common cause of SCD in young athletes [2]. It is still a frequently neglected topic in cardiology. Autopsy series reveals that the majority of cases concern young patients (<35 years old) who die during or shortly after exercise [3] but can go unnoticed in the elderly patients until they present with myocardial infarction due to coronary atherosclerosis [4].

So far, there is no imaging test of choice for the diagnosis of AAOCA. When a coronary anomaly is assessed, nonpharmacological functional imaging such as echocardiography, nuclear study, or cardiovascular magnetic resonance with physical stress are recommended to be performed to measure myocardial ischemia (*class I* recommendation) [3].

There are multiple variants of AAOCA, and some of them are associated with a higher risk for SCD. AAOCA can be classified based on (i) the origin of the anomalous vessel: left coronary artery, right coronary artery, single coronary artery, or the circumflex coronary artery; (ii) the course of the anomalous vessel: intramural, intra-arterial, intramyocardial, prepulmonic, subpulmonic, and retroaortic; and (iii) the location, shape, and spatial relationship of the coronary ostia: two separate nonconfluent orifices arising from the same sinus, two confluent orifices located adjacent to each other, or one single orifice with bifurcation in the aortic wall and single orifice with bifurcation outside the aortic wall [5].

The exact prevalence of AAOCA in the general population, as well as the associated risk of SCD, remains unknown [6]. The prevalence of AAOCA in angiographic series has been reported between 0.6% and 1.3% and 0.3% in autopsies [7, 8]. In a prospective study by Yamanaka et al. based on 126,595 patients with coronary artery anomalies, 80% were benign and asymptomatic. However, 20% were life-threatening and presented with arrhythmia, syncope, myocardial infarction, or sudden cardiac death [9].

In our case report, the patient presented an anomaly of the right coronary artery (ARCA) with an interarterial course as well as an intramural course arising just above the contralateral sinus of Valsalva. Of 8,522 patients referred for coronary computed tomography angiography, 72 (0.84%) had an anomalous coronary artery from opposite sinus of Valsalva, including right-sided origins of the left main coronary artery, left anterior descending coronary artery, left circumflex coronary artery, and left-sided origin of the right coronary artery [10]. In the latter study, 20 patients (0.22%) had left-sided origin of the RCA. Among patients with an anomalous coronary artery from the opposite sinus of Valsalva, 23 patients (0.28%) had an interarterial path. In this group, 6 patients (0.07%) had a high-risk course, defined in the review by a slit-like ostium, an acute angle take-off, an intramural route, and a significant compression between the aorta and the pulmonary trunk existing simultaneously. These high-risk features of an anomalous coronary artery from opposite sinus of Valsalva were only found in case of left-sided right coronary arteries, as was the case in our patient. Moreover, intra-arterial course is more frequent in ARCA, when compared to anomalous left coronary artery (0.23% versus 0.03%, respectively) [6].

The European Society of Cardiology's (ESC) identified risk factors for myocardial ischemia, such as high orifice, ostial stenosis, slit-like/fish-mouth-shaped orifice, acute angle take-off, intramural course and its length, interarterial path, and hypoplasia of the proximal coronary artery [3]. Our case presented several of these features, such as the hypoplastic ostium of the RCA, the interarterial course between the aorta and the pulmonary artery, and the short intramural path. Moreover, symptomatic patients such as the case we report is a *class I* recommendation for surgery [3]. Additionally, ischemia of 10% in the territory of the RCA found during PET-CT put him at higher risk for SCD.

Current guidelines do not support screening for AAOCA in asymptomatic athletes, given the low prevalence of this disease [6]. Further studies and clarification on the screening options of athletes remain an issue. Currently, the ESC and The American Heart Association and American Academy of Pediatrics both provide a recent consensus statement on cardiovascular screening of young college athletes [11, 12].

Surgery is indicated in case of symptomatic patients with left-sided origin of the right coronary artery with interarterial, intramural path and with hypoplasic ostia. Nevertheless, management of critical patients with intra-arterial course of an anomalous coronary artery is controversial.

Physicians should be aware of the symptoms of AAOCA, as these rare congenital abnormalities can lead to death. Appropriate imaging studies should be performed, and cases should be discussed with a multidisciplinary team.

## Data Availability

The data used to support the findings of this study are included within the article.
